# Genome and Phylogenetic Analysis of Infectious Hematopoietic Necrosis Virus Strain SNU1 Isolated in Korea

**DOI:** 10.3390/pathogens8040200

**Published:** 2019-10-21

**Authors:** Woo Taek Oh, Jin Woo Jun, Sib Sankar Giri, Saekil Yun, Hyoun Joong Kim, Sang Guen Kim, Sang Wha Kim, Se Jin Han, Jun Kwon, Se Chang Park

**Affiliations:** 1 Laboratory of Aquatic Biomedicine, College of Veterinary Medicine and Research Institute for Veterinary Science, Seoul National University, Seoul 08826, Korea; mike0202@snu.ac.kr (W.T.O.); giribiotek@gmail.com (S.S.G.); arseidon@snu.ac.kr (S.Y.); hjoong@snu.ac.kr (H.J.K.); imagine0518@snu.ac.kr (S.G.K.); kasey.kim90@gmail.com (S.W.K.); sejin.n@snu.ac.kr (S.J.H.); kjun1002@snu.ac.kr (J.K.); 2 Department of Aquaculture, Korea National College of Agriculture and Fisheries, Jeonju 54874, Korea; advancewoo@snu.ac.kr

**Keywords:** IHNV, Korean isolates, phylogenetic analysis, genome sequencing, trout fisheries

## Abstract

Infectious hematopoietic necrosis virus (IHNV), one of the most important pathogenic fish viruses, affects trout fisheries and causes considerable economic losses. Currently, in Korea, more studies on IHNV infection are being reported. However, relatively less data is available on Korean isolates than on those from other countries. Few studies have focused on gene sequence analyses of IHNV glycoprotein (*G*) gene and almost none have focused on other gene fragments. Therefore, considering the dearth of adequate phylogenetic and genomic studies on Korean IHNV strains because of the lack of data, our study aimed to provide sufficient relevant data by sequencing the complete genome of the IHNV strain SNU1, which was recently isolated from a Korean rainbow trout farm. Moreover, we focused on expanding the perspectives on the phylogenesis of IHNV isolates from Korea and other Asian countries. IHNV was isolated from pooled hematopoietic tissue samples using *Epithelioma papulosum cyprinid* (EPC) cells, and phylogenetic analysis and genome study were conducted using complete *G*, *N*, and nonvirion (*NV*) gene sequences. Our main achievements were the development of a phylogenetic analytical method based on the *NV* gene and complete genome sequence analysis of the IHNV strain SNU1, which was compared with other Asian isolate sequences.

## 1. Introduction

Infectious hematopoietic necrosis virus (IHNV), one of the most devastating fish pathogens, causes considerable economic losses to the aquaculture industry [1]. The virus is a single-stranded, bullet-shaped, ribonucleic acid (RNA) virus that belongs to the genus *Norvirhabdovirus* and the family *Rhabdoviridae* [2]. The virus was first reported in the 1950s in sockeye salmon (*Oncorhynchus nerka*) from Washington and Oregon fish hatcheries in the United States [3]. Diverse fish species are known to be susceptible to IHNV infections including salmonids, which are the most vulnerable [4]. The pathogenicity of the virus is known to depend on environmental conditions and the size and species of the fish [1]. Outbreaks of IHNV infection are usually associated with a mortality rate of 50–60%, but can be up to 95%, and younger fish have been found to be more susceptible to the clinical symptoms and acute infections [1,5]. The virus was first reported in Asian countries in 1971 when diseased kokanee salmon (freshwater *O. nerka*) were imported to Japan from North America [6]. Although the consumption amount of salmonids and the size of the fisheries rearing this species in Korea are relatively smaller than those in other countries in Europe and North America, some previous studies have been conducted on the presence of IHNV in Korea. In 1991, the first outbreak of IHNV was reported in Korea in cultured rainbow trout (*Oncorhynchus mykiss*) and masuo salmon (*Oncorhynchus masuo*) [7]. 

The IHNV genome consists of six genes in the order 3’-N-P-M-G-NV-L-5’, representing the nucleocapsid protein (N), phosphoprotein (P), matrix gene (M), glycoprotein (G), nonvirion protein (NV), and polymerase protein (L) [8]. The size of the genome was approximately 11 kb, and studies analyzing the genome, phylogenesis, and diversity of IHNV have usually used the *G*, *N*, and *NV* genes [4]. Further, epidemiologic studies, including phylogenetic analyses of IHNV isolated from different countries worldwide, have identified five genogroups to date [9]. The presence of genogroups U, M, and L was reported in North America based on studies of isolates collected from genographic areas in the Pacific Northwest of North America including Alaska, British Columbia, the Columbia River basin, and Idaho. Moreover, the genogroup L was observed in isolates from California and the southern Oregon coast [10]. The remaining two genogroups are the E and J genogroups. The lineage of the E genogroup is European, and it is divided into two subgroups: E1 and E2. The J genogroup represents isolates from Japan and South Korea and is also divided into two subclades: J Nagano (JN) and J Shizuoka (JS) [6]. In addition, a recent study phylogenetically analyzing Chinese isolates based on the *G* gene reported that Chinese IHNV isolates form another individual clade belonging to the J genogroup with Asian lineage [11,12].

IHNV is one of the most well-known viruses that cause significantly high mortality rates in rainbow trout fisheries, and recent research indicates that reports on IHNV infections are increasing, especially in almost all geographic regions of China [13]. Moreover, compared to before 2000, the number of IHNV isolates has gradually increased in Korea recently [5]. No study has reported the complete genome sequence of the IHNV isolates in Korea, and the quality of data associated with the phylogenetic analysis of the Korean IHNV isolates is poor compared with that of the data of studies conducted in other countries. Therefore, our study aimed to provide sufficient information on the phylogenetics of IHNV isolates from Korea. Further, to the best of our knowledge, this is first study to report the complete genomic sequence of the IHNV strain isolated from Korea. In addition, we also aimed to study the nucleotide substitution and phylogenetic patterns of IHNV isolated from Korea and compared them to those of other isolates from Asia, Europe, and North America.

## 2. Material and Methods

### 2.1. Sample Collection, Examination, and Extraction of Viral RNA 

In May 2019, mass mortality was observed in one of the rainbow trout farms located in the Kangwon province, South Korea. The farm contained six water tanks with open circulating systems that were supplemented with 4.5 tons of ground water. The rearing size of the rainbow trout fry was 3–7 g, and 50,000 fish were kept in each water tank. The dissolved oxygen in each water tank was measured and sustained at the ideal range required for the optimal growth conditions of the fish, and the water temperature was maintained at 12–13 °C. The cumulative mortality rate in four days was approximately 85%, and the moribund fish were sent to the Laboratory of Aquatic Biomedicine, Seoul National University, South Korea to determine the causative agent responsible for the disease. The fish were inspected for viral, bacterial, fungal, and parasitic infections. For the fungal and parasitic examinations, the fins and gills of the fish sent for diagnosis were first macroscopically observed. Subsequently, each part of the fins and gills were swabbed, smeared on glass slides, and microscopically examined under a light microscope. For the internal organ examination, the kidneys, liver, and spleen of the fish were visually inspected and separated for diagnosis. For the diagnosis of potential bacterial infection, the separated internal organs were homogenized in 300 µL phosphate-buffered saline (PBS), and 100 µL of the homogenate was inoculated onto tryptic soy agar (TSA). The agar plates were streaked and incubated at 20 °C and 25 °C for 48 h and were then inspected for the presence of bacterial isolates. To detect potential viral infections, the viral RNA was extracted from the spleen and kidney tissues from the moribund fish using the Viral Gene-spin^TM^ viral DNA/RNA extraction kit (iNtRON Biotechnology, Daejeon, Korea) based on the manufacturer’s protocol. Tests to detect three major viral agents, infectious pancreatic necrosis virus (IPNV), viral hemorrhagic septicemia virus (VHSV), and IHNV, which affect rainbow trout, were performed using polymerase chain reaction (PCR) tests with specific primers following the same conditions reported in previous studies [14,15,16]. In addition, for any possible mortality causing agents excluding bacteria, virus, and parasites such as rickettsial infection, detection of *Piscirickettsia salmonis* and rickettsia-like organisms inducing red mark syndrome in rainbow trout were performed using PCR with specific primers [17,18].

### 2.2. Virus Isolation and Complete Sequencing

For the isolation of IHNV, pooled samples of hematopoietic tissues (the kidney, spleen, and liver) were homogenized in PBS, and the virus was isolated using the *Epithelioma papulosum cyprinid* (EPC) cell line. Cells were grown in minimum essential medium (MEM, Difco, Sparks, USA) supplemented with 10% fetal bovine serum and antibiotics (penicillin 100 IU/mL). The homogenized tissue solutions were centrifuged at 13,000 rpm for 10 min, and the supernatant was filtered using a 0.2 µm filter, inoculated on cell monolayers grown on cell culture plates, and incubated at 15 °C for four days. Then, the cytopathic effect (CPE) was determined on the monolayers of cells, and for the adaptation and amplification of the virus, the same process was repeated with cell culture passage five. The supernatants of the monolayers displaying the CPE were frozen at −80 °C and the same procedure was repeated with the viral RNA extract. Viruses were identified using PCR with specific primers. In addition, and for the complete sequencing process, complementary DNA (cDNA) was synthesized using the PrimeScript^TM^ 1st Strand cDNA synthesis kit (TaKaRa, Shiga, Japan). For the complete sequencing of the virus, 13 pairs of primers were used, including six pairs of specific primers designed for this study and seven other pairs based on a previous study protocol [13] using the same PCR conditions. The primers and PCR conditions used in this study are presented in Table 1. The complete genomic sequence at the 5’ and 3’ ends were identified using the same procedure reported in a previous study [13]. Specific PCR bands were purified using the QIAquick gel extraction kit (Qiagen, CA, USA), cloned using the TA cloning kit (Topcloner TA kit; Enzynomics, Daejeon, Korea), and subsequently transformed into competent *Escherichia coli* cells (DH5α). The purified recombinant plasmids were then sequenced by the Genomic Division of Macrogen (Seoul, Korea), where the nucleotide sequencing reaction was performed using the ABI PRISM 3730XL analyzer with BigDye ® terminator v3.1 cycle sequencing kits (Applied Biosystems, MA, USA). The complete sequence was analyzed using the Lasergene software (DNAStar, WI, USA), which determined the percentage identities and distances to calculate the similarity scores and protein translation and multiple alignments. The default options in Clustal W were used for the alignment of whole sequences [11].

### 2.3. Phylogenetic Analysis 

For the phylogenetic analysis, the complete sequences of the *G*, *N*, and *NV* genes were collected from GenBank server and were aligned with the IHNV strain, SNU1 (Accession number: MN225937) using the BioEdit software, version 7.0.9 [19]. We used 120, 41, and 31 sequences (in the order of *G*, *N*, and *NV* genes) for the construction of phylogenetic trees. The phylogenetic trees were constructed using maximum likelihood method of MEGA 10.0 software with a bootstrap number of 1000 replicates. 

### 2.4. Analysis of Single Nucleotide Polymorphisms

The G protein of 40 Asian IHNV isolates (9 Korean, 18 Japanese, and 13 Chinese isolates) were collected (Table S2) to analyze the distribution of single nucleotide polymorphisms (SNPs) in the Asian IHNV strains. An almost complete G protein (1423 bp) fragment, excluding the trimmed primer regions, was used for the study, and the alignments were checked manually in to confirm the absence of existing artefactual gaps. The Lasergene software (DNAStar, WI, USA) was used for multiple alignment of the sequences, and the SeqMan Pro^TM^ software was used for the variant nucleotide check. The rest of the isolates were compared using the SNU1 strain as a reference strain, starting from the consensus sequence at each nucleotide site along the 1423 nt *G* gene. The percentage of strains displaying the variant nucleotide sites was calculated and is depicted in graph form.

## 3. Results and Discussion

### 3.1. Sample Examination and Virus Isolation

The rapid and high mortality rate of the investigated disease suggested that the causative agent might be a virus rather than a bacterium or parasite. Further, the size and aquatic environment of the diseased fish aligned perfectly with those associated with viral infections, and the sizes of the diseased fish were <7 g [20]. A visual observation of the fins and gills of the moribund fish clearly indicated that they were not affected by any parasitic or fungal infection. Additionally, the results of microscopic inspections to detect bacterial and parasitic infections were also negative. The internal organs of the diseased fish exhibited several typical clinical symptoms of a viral infection, such as splenomegaly and hemorrhage [1]. The gills of most of the fish that were sent for diagnosis exhibited an anemic appearance, and a few of the fish also displayed abdominal distension. During the bacterial isolation, no specific colonies appeared to grow on the TSA plates incubated at both 20 °C and 25 °C. In addition, the PCR analysis to identify any rickettsial infections was negative. Regarding the viral infection tests, the PCR assay conducted using IHNV specific primers [14] targeting the partial *G* gene fragment yielded a positive result, and the results of the IPNV and VHSV tests were both negative. Therefore, IHNV was isolated using an EPC cell line, and we began to observe CPE after two days of incubation at 15 °C (Figure S1). For amplification of the bacteria, cell culture plates from the fifth passage were used for viral RNA extraction. The outcomes of the bacterial isolation were double-checked using RNA extracted from the supernatant of the fifth cell culture plate using a PCR assay with specific primers that generated clear, positive results. 

### 3.2. Complete Sequence of the IHNV Strain SNU1 

There is a dearth of research on the complete sequencing of IHNV isolated in Korea, and molecular characterization has most frequently been performed only using the *G* viral gene. To uncover the complete genomic sequence of the virus, we used the same sequencing procedure reported in a previous study using seven primer pairs, and we uncovered a 7 kb (3767–10,993)-long partial sequence. However, the PCR results of the analysis of the remaining parts of the sequence were found to be negative. Therefore, specific primers were designed to target the rest of the gene fragments (Table 1), and by securing the fragment sequence using the SMART^TM^ rapid amplification of the cDNA ends (RACE) cDNA amplification kit (TaKaRA, Shiga, Japan), the complete sequence of the SNU1 strain was obtained.

The complete sequence of the SNU1 strain was compared to those of seven isolates that were completely sequenced and registered in the GenBank server (Accession number: L40883, JX649101, KJ521216, MH374162, MF509592, GQ413939, X89213, and MN225937). The similarity values ranged from 95.0% to 96.6% and the distance value calculated using the Lasergene software indicated that the SNU1 strain was closely related to the IHNV strains, HLJ-09 and BJSY, which were both isolated from China (distance value: 0.03, 0.05). The similarity values of eight IHNV strains are provided in Supplementary Table S1. Moreover, the *G* gene of the SNU1 strain was compared to those of other Asian isolates using the same method. The resulting similarity values varied from 99.3% to 99.9% with the following calculated distance scores: 0.029 (RtNAG96), 0.017 (RtGoH14), 0.016 (PcKw11), and 0.023 (RtUi02), and the four highest scores implied that the strain was highly similar to those of other Korean isolates rather than those of other Asian isolates. The genetic diversity of the Korean isolates was smaller than that of the Chinese isolates since the similarity values of 42 Chinese isolates ranged from 98.0% to 100.0% [11].

### 3.3. Phylogenetic Analysis of IHNV Strain SNU1

**Figure 1:**
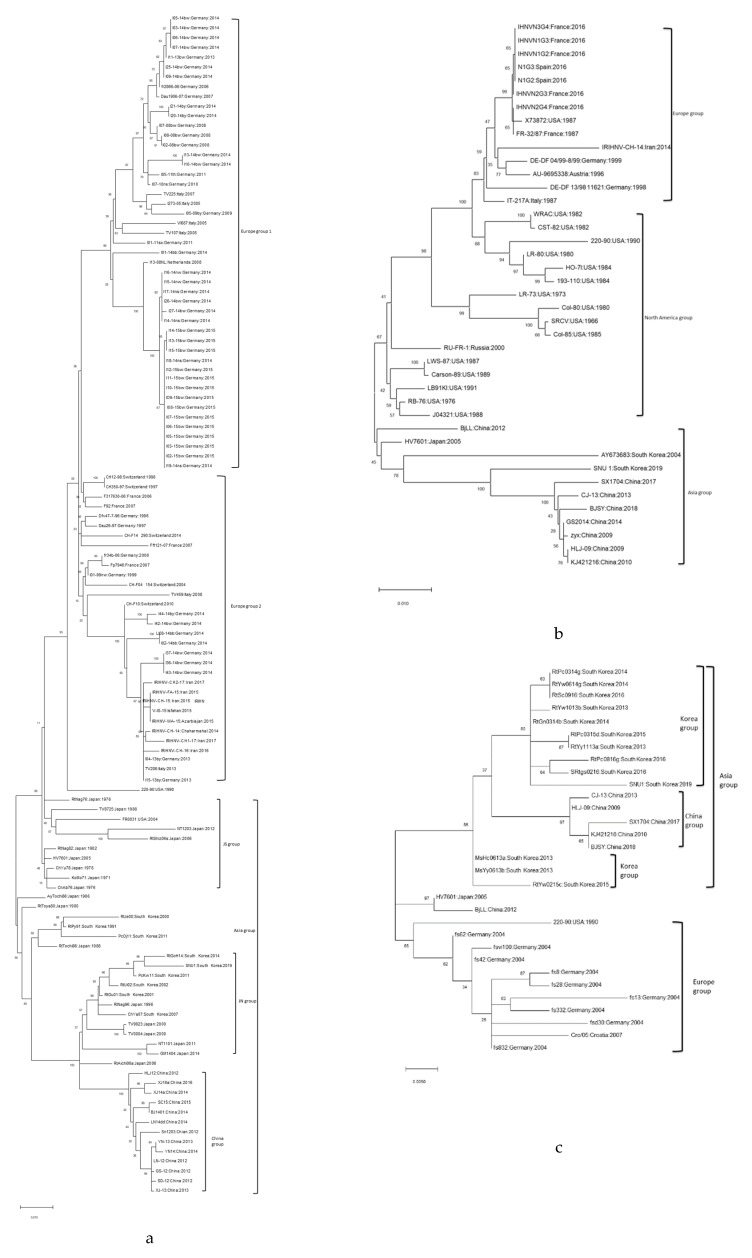
Phylogenic trees created with the glycoprotein (*G*) (**a**), nucleocapsid protein (*N*) (**b**), and nonvirion (*NV*) (**c**) gene sequences. The scale axis indicates substitutions per site and the tree was constructed using maximum likelihood method of MEGA 10.0.

The results of the phylogenetic analysis performed using the *G* gene (Figure 1a) revealed that the SNU1 strain was included in the JN group with other South Korean isolates. The phylogenetic tree constructed based on the *N* gene (Figure 1b) yielded a similar result, indicating that the group was divided into two subclades, with one including both the European and the North American isolates and the other including the Asian isolates. Similar to the results of the *G* and *N* genes, the phylogenetic tree results of the *NV* gene (Figure 1c) also yielded a similar outcome, but there was a distinct trait associated with the Asian lineage group. Further, the Asian isolates were divided into three groups: A Chinese group, South Korean group, and Chinese and Japanese combined group, which indicated that the *NV* gene of the isolates from South Korea formed a clade that distinguished it from the isolates of the other Asian countries. 

### 3.4. Features of IHNV Korean Isolates

**Figure 2:**
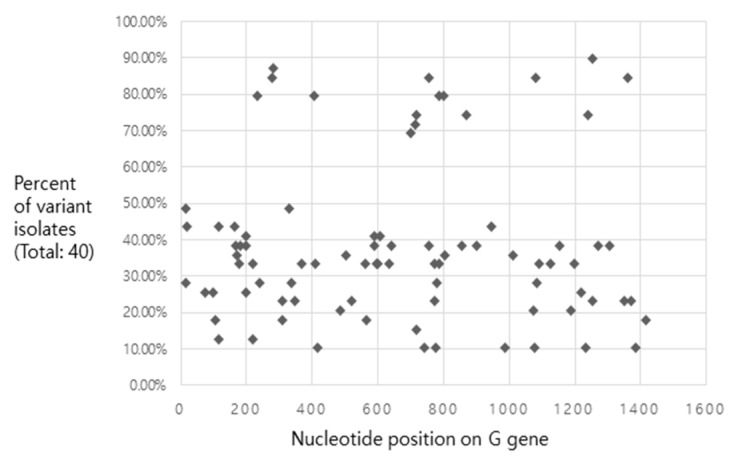
Single nucleotide polymorphisms (SNPs) along the *G* gene of 40 Asian isolates. The reference genome was designated as the SNU1 strain, and the distribution of the nucleotide variant isolates is expressed as percentage.

To measure the variant nucleotide distribution of the Asian IHNV isolates, 40 *G* genes were collected and aligned using the analytical method of the SeqMan Pro^TM^ software (Figure 2). 

The results of the analysis of variance in the SNPs indicated that there may be a common nucleotide position that distinguishes the isolates based on the country of origin, since the percentage of variants was estimated to be <50% or >70%, and the values from 50 to 70 were clear. Detailed information on the distribution is presented in Table S3. Additionally, the aforementioned genes were translated into protein sequences, and in some specific codon positions, the Asian isolates could be distinguished from each other based on their country of origin. The isolates from Japan exhibited a distinct protein sequence at positions 66, 103, 112, 361, and 450 compared to the isolates from Korea and China. In the Chinese isolates, proteins at positions 168, 187, 211, and 423 exhibited distinct features from those of isolates from Korea and Japan (Figure 2). The following *G* genes were analyzed to calculate the distance between the isolates from Asia using the Lasergene software, with the same method used for distance calculation between complete IHNV sequences. The minimum and maximum distance scores of the Asian isolates were 99.92 and 99.99, respectively. Details of the values are presented in Table S4. 

Diverse approaches have been used in the evolutionary analysis and phylogenetic studies of salmonids viruses, such as IHNV, IPNV, and VHSV, using deep sequence data or evolutionary dynamics methods [11,21,22,23]. Our study aimed to analyze the sequences of the Korean IHNV isolates and compare them with those of IHNV strains from other countries, especially other Asian isolates. Since this is, to the best of our knowledge, the first research study to report the complete sequence of IHNV isolated from Korea, the similarities among the entire IHNV genome and those of other strains isolated from Korea cannot be compared. However, the research results indicated that the SNU1 strain was highly similar (96.6%) to the HLJ-09 strain isolated from China [24]. The differences in the similarity values between the Korean and Chinese isolates were not low compared to the differences in these values of isolates from China [11,13]. Moreover, the distance values calculated based on the *G* gene of the IHNV isolates indicated that the SNU1 strain was closely related to strains from other Korean isolates with distance scores ranging from 0.01 to 0.03. Further, there are no previous reports on SNP distribution analyses conducted on Asian isolates, and our study focused on identifying the distinct characteristics of the nucleotide and protein sequences based on the countries of origin of the isolates. 

The phylogenetic study results were similar to those of previous studies that divided the IHNV genogroups into five groups based on an analysis of the *G* gene. The SNU1 strain was also included in the genogroup JN, which aligned with the expected result of where it would have been included in either the JN or JS genogroups in previous studies conducted in Korea [5]. However, our study suggested that IHNV isolates could also be classified into distinct genogroups based on the *NV* gene apart from the *G* gene. Moreover, phylogenetic studies based on the *NV* gene yielded clearer results for the Korean isolates, since the subclade consisting of only Korean isolates was distinguished from the clade consisting of other Asian isolates. The phylogenetic analyses in previous studies usually focused on variances of the *G* gene, which they used to determine genetic diversity. Therefore, the IHNV isolates from Asia were only divided into two groups, but in our study, we easily distinguished the Chinese, Japanese, and South Korean clades based on the phylogenetic analysis of the *NV* gene. Additionally, the results of the SNP variant analyses indicated that the Asian isolates had distinct characteristics depending on their country of origin.

## 4. Conclusions

Studies focused on the genome analysis of IHNV isolates have been conducted using various methods in other countries, but not for Korean isolates. Since the occurrence rate of IHNV infection has increased in the 21st century in Korea, the need for a study focused on the phylogenesis and evolution of Korean isolates is urgent to develop a vaccine that can be used to protect rainbow trout fish in Korea. Currently, the vaccines for the prevention of IHNV are widely studied globally [25]. Therefore, an effective vaccine is also needed for Korean aquaculture, which was one of the aims of our study in investigating the IHNV. Some of our new findings on phylogenetic analyses based on the *NV* gene and the complete sequence of the Korean isolate would facilitate the enlargement of perspectives of other future studies focused on the phylogenesis and genotypic distribution of the virus. 

## Figures and Tables

**Table 1:** Polymerase chain reaction (PCR) conditions and primers designed to sequence the infectious hematopoietic necrosis virus (IHNV) gene fragments in this study.

IHNV Primer	Sequences (5’-3’)	Position	Annealing Temp, Time
IHNV 1FF	GTATAAGAAAAGTAACTTGACT	1	50 °C, 30 s
IHNV 1RR	CAATCCCTTGGCTGGTTGC	670	
IHNV 2FF	CAAGCTCGAGGTCTTGCAA	610	55 °C, 30 s
IHNV 2RR	CCCTCTCCGGTTGAGCCAT	1220	
IHNV 3FF	GAGATCGCTCGTCTCCTTGT	1160	50 °C, 30 s
IHNV 3RR	GGCCTGGTGGGCCTGTCT	1760	
IHNV 4FF	GAGAGCTGTCAGGATGCC	1690	54 °C, 30 s
IHNV 4RR	CCTCTCCTCGTCTCCGCT	2330	
IHNV 5FF	CAAACGAGAGCATGTCTATTTTCA	2240	55 °C, 30 s
IHNV 5RR	TTTGGCTGTTTGCTCCGCAG	3060	
IHNV 6FF	CCTGTTTTCATCCAGCCATGT	2690	55 °C, 60 s
IHNV 6RR	CCTCAAGACATTCCTCTCTG	3940	

## Supplementary Materials

The following are available online at https://www.mdpi.com/2076-0817/8/4/200/s1, Figure S1: CPE result of EPC cell line after 48h infection with IHN virus strain SNU1. (Bar indicating 50 μm), Table S1: Similarity values calculated between eight complete sequences of IHNV using MegAlign pro 16, Table S2: Information about strains used for the analysis of distribution in single nucleotide SNPs of 40 Asian isolates, Table S3: Distribution of SNPs of 40 Asia isolates compared with strain SNU1 as a reference, Table S4: Distance scores calculated using 40 Asia isolates by SeqMan Pro^TM^ software.
