# Supplementary material for: Genome and Phylogenetic Analysis of Infectious Hematopoietic Necrosis Virus Strain SNU1 Isolated in Korea
Source: Pathogens. 2019 Oct 21;8(4):200. doi: 10.3390/pathogens8040200 (PMC6963739; doi:10.3390/pathogens8040200)
Supplement: Supplementary file 1 [file pathogens-08-00200-s001.pdf]

pathogens

MDPI

**Table S1**. Similarity values calculated between eight complete sequences of IHNV using MegAlign pro 16.

| | L40883 | JX649101 (HLJ-09) | KJ421216 | MH374162 (BJSY) | MF509592 (BJLL) | GQ413939 (220-90) | X89213 | MN 225937 (SNU1 this study) |
|---|---|---|---|---|---|---|---|---|
| L40883 | - | 95.5 | 95.5 | 95.3 | 97.4 | 97.2 | 98.2 | 95.0 |
| JX649101 (HLJ-09) | 95.5 | - | 99.8 | 99.7 | 96.4 | 95.0 | 95.9 | 96.6 |
| KJ421216 | 95.5 | 99.8 | - | 99.8 | 96.4 | 95.0 | 95.9 | 96.6 |
| MH374162 (BJSY) | 95.3 | 99.7 | 99.8 | - | 96.2 | 94.9 | 95.7 | 96.5 |
| MF509592 (BJLL) | 97.4 | 96.4 | 96.4 | 96.2 | - | 96.7 | 97.9 | 95.8 |
| GQ413939 (220-90) | 97.2 | 95.0 | 95.0 | 94.9 | 96.7 | - | 97.5 | 94.6 |
| X89213 | 98.2 | 95.9 | 95.9 | 95.7 | 97.9 | 97.5 | - | 95.4 |
| MN 225937 (SNU1 this study) | 95.0 | 96.6 | 96.6 | 96.5 | 95.8 | 94.6 | 95.4 | - |

**Table S2.** Information about strains used for the analysis of distribution in single nucleotide SNPs of 40 Asian isolates.

| Strain Name | Origin (Isolated Country) | Isolated Year | Length (bp) |
|---|---|---|---|
| XJ-13 | China | 2013 | 1527 |
| SD-12 | China | 2012 | 1527 |
| GS-12 | China | 2012 | 1527 |
| YN14 | China | 2014 | 1527 |
| YN-13 | China | 2013 | 1527 |
| HLJ12 | China | 2012 | 1527 |
| XJ14a | China | 2014 | 1527 |
| XJ16a | China | 2016 | 1527 |
| HV7601 | Japan | 2005 | 1674 |
| KoMo71 | Japan | 1971 | 1527 |
| RtNag82 | Japan | 1982 | 1527 |
| ChAb76 | Japan | 1976 | 1527 |
| ChYu78 | Japan | 1978 | 1527 |
| RtNag76 | Japan | 1976 | 1527 |
| TV8725 | Japan | 1988 | 1527 |
| RtShiz06s | Japan | 2006 | 1485 |
| NT1203 | Japan | 2012 | 1527 |
| AyTochi86 | Japan | 1986 | 1527 |

| | | | |
|---|---|---|---|
| RtToya80 | Japan | 1980 | 1527 |
| RtTochi86 | Japan | 1986 | 1527 |
| RtPy91 | South Korea | 1991 | 1527 |
| RtJe00 | South Korea | 2000 | 1527 |
| PcOj11 | South Korea | 2011 | 1536 |
| RtAichi06a | Japan | 2006 | 1485 |
| TV0004 | Japan | 2000 | 1527 |
| TV9923 | Japan | 2000 | 1527 |
| RtNag96 | Japan | 1996 | 1527 |
| SNU1 | South Korea | 2019 | 1527 |
| RtGoH14 | South Korea | 2014 | 1423 |
| PcKw11 | South Korea | 2011 | 1527 |
| RtUi02 | South Korea | 2002 | 1527 |
| RtGu01 | South Korea | 2001 | 1527 |
| ChYa07 | South Korea | 2007 | 1527 |
| GM1404 | Japan | 2014 | 1527 |
| NT1101 | Japan | 2011 | 1527 |
| BJ1401 | China | 2014 | 1527 |
| SC15 | China | 2015 | 1527 |
| Sn1203 | China | 2012 | 1527 |
| LN14dd | China | 2014 | 1527 |
| LN-12 | China | 2012 | 1527 |

**Table S3.** Distribution of SNPs of 40 Asia isolates compared with strain SNU1 as a reference.

| Nucleotide Position | Reference Position | Reference Base | Called Base | SNP % | A Cnt | C Cnt | G Cnt | T Cnt |
|---|---|---|---|---|---|---|---|---|
| **16** | 16 | C | A | 28.20% | 10 | - | 0 | 1 |
| **17** | 17 | C | A | 48.71% | 19 | - | 0 | 0 |
| **18** | 18 | A | C | 43.58% | - | 17 | 0 | 0 |
| **75** | 75 | C | T | 25.64% | 0 | - | 0 | 10 |
| **99** | 99 | A | G | 25.64% | - | 0 | 10 | 0 |
| **105** | 105 | C | T | 17.94% | 1 | - | 0 | 6 |
| **114** | 114 | T | C | 12.82% | 0 | 5 | 0 | - |
| **117** | 117 | T | C | 43.58% | 0 | 17 | 0 | - |
| **165** | 165 | G | A | 43.58% | 16 | 0 | - | 1 |
| **169** | 169 | T | G | 38.46% | 0 | 0 | 15 | - |
| **171** | 171 | C | T | 35.89% | 0 | - | 0 | 14 |
| **178** | 178 | A | G | 33.33% | - | 0 | 13 | 0 |
| **180** | 180 | C | A | 38.46% | 15 | - | 0 | 0 |
| **197** | 196 | G | T | 38.46% | 1 | 0 | - | 14 |
| **198** | 197 | C | T | 41.02% | 0 | - | 0 | 16 |
| **199** | 198 | G | A | 25.64% | 10 | 0 | - | 0 |
| **219** | 218 | C | A | 33.33% | 13 | - | 0 | 0 |
| **220** | 219 | G | T | 12.82% | 0 | 1 | - | 4 |
| **232** | 231 | G | A | 79.48% | 31 | 0 | - | 0 |
| **239** | 238 | T | C | 28.20% | 0 | 11 | 0 | - |
| **277** | 276 | C | A | 84.61% | 32 | - | 0 | 1 |
| **283** | 282 | T | C | 87.17% | 0 | 34 | 0 | - |
| **309** | 308 | T | C | 23.07% | 0 | 9 | 0 | - |
| **310** | 309 | G | A | 17.94% | 7 | 0 | - | 0 |
| **331** | 330 | T | A | 48.71% | 13 | 6 | 0 | - |
| **336** | 335 | G | A | 28.20% | 11 | 0 | - | 0 |
| **349** | 348 | A | G | 23.07% | - | 1 | 8 | 0 |
| **367** | 366 | A | G | 33.33% | - | 0 | 13 | 0 |
| **406** | 405 | T | C | 79.48% | 0 | 31 | 0 | - |
| **409** | 408 | C | T | 33.33% | 1 | - | 0 | 12 |
| **418** | 417 | A | G | 10.25% | - | 0 | 4 | 0 |
| **487** | 486 | T | C | 20.51% | 0 | 8 | 0 | - |
| **503** | 502 | A | G | 35.89% | - | 0 | 14 | 0 |
| **520** | 519 | T | C | 23.07% | 0 | 9 | 0 | - |
| **562** | 561 | G | T | 33.33% | 0 | 0 | - | 13 |
| **565** | 564 | T | C | 17.94% | 0 | 5 | 2 | - |

| | | | | | | | | |
|---|---|---|---|---|---|---|---|---|
| **589** | 588 | C | T | 38.46% | 0 | - | 0 | 15 |
| **590** | 589 | G | T | 41.02% | 1 | 0 | - | 15 |
| **597** | 596 | C | A | 33.33% | 13 | - | 0 | 0 |
| **601** | 600 | G | A | 33.33% | 13 | 0 | - | 0 |
| **607** | 606 | C | A | 41.02% | 15 | - | 0 | 1 |
| **636** | 635 | A | G | 33.33% | - | 0 | 13 | 0 |
| **642** | 641 | C | T | 38.46% | 0 | - | 0 | 15 |
| **701** | 700 | A | G | 69.23% | - | 0 | 27 | 0 |
| **715** | 713 | A | G | 71.79% | - | 0 | 28 | 0 |
| **716** | 714 | A | G | 74.35% | - | 0 | 29 | 0 |
| **717** | 715 | C | A | 15.38% | 3 | - | 0 | 2 |
| **740** | 738 | T | C | 10.25% | 0 | 4 | 0 | - |
| **755** | 753 | A | C | 38.46% | - | 15 | 0 | 0 |
| **757** | 755 | G | A | 84.61% | 16 | 3 | - | 14 |
| **771** | 769 | G | T | 23.07% | 0 | 0 | - | 9 |
| **774** | 772 | A | G | 33.33% | - | 0 | 13 | 0 |
| **775** | 773 | A | T | 10.25% | - | 1 | 0 | 3 |
| **778** | 776 | C | T | 28.20% | 0 | - | 0 | 11 |
| **785** | 783 | G | T | 33.33% | 0 | 2 | - | 11 |
| **788** | 786 | T | C | 79.48% | 0 | 31 | 0 | - |
| **800** | 798 | T | G | 79.48% | 0 | 0 | 31 | - |
| **804** | 802 | A | G | 35.89% | - | 0 | 14 | 0 |
| **854** | 852 | C | T | 38.46% | 0 | - | 0 | 15 |
| **869** | 867 | G | T | 74.35% | 14 | 0 | - | 15 |
| **899** | 897 | C | T | 38.46% | 0 | - | 0 | 15 |
| **944** | 942 | T | C | 43.58% | 0 | 17 | 0 | - |
| **986** | 984 | T | C | 10.25% | 0 | 3 | 1 | - |
| **1010** | 1008 | C | T | 35.89% | 0 | - | 0 | 14 |
| **1075** | 1073 | A | G | 20.51% | - | 0 | 6 | 2 |
| **1076** | 1074 | C | T | 10.25% | 0 | - | 0 | 4 |
| **1079** | 1077 | A | C | 84.61% | - | 19 | 0 | 14 |
| **1084** | 1082 | A | G | 28.20% | - | 0 | 11 | 0 |
| **1091** | 1089 | G | T | 33.33% | 0 | 0 | - | 13 |
| **1124** | 1122 | G | A | 33.33% | 13 | 0 | - | 0 |
| **1153** | 1151 | T | C | 38.46% | 0 | 15 | 0 | - |
| **1187** | 1185 | C | A | 20.51% | 6 | - | 0 | 2 |
| **1198** | 1196 | T | C | 33.33% | 0 | 13 | 0 | - |
| **1218** | 1216 | A | G | 25.64% | - | 0 | 10 | 0 |

| | | | | | | | | |
|---|---|---|---|---|---|---|---|---|
| **1232** | 1230 | A | G | 10.25% | - | 2 | 2 | 0 |
| **1241** | 1239 | G | A | 74.35% | 27 | 2 | - | 0 |
| **1252** | 1250 | A | C | 89.74% | - | 34 | 0 | 1 |
| **1254** | 1252 | T | C | 23.07% | 0 | 9 | 0 | - |
| **1271** | 1269 | C | A | 38.46% | 13 | - | 0 | 2 |
| **1304** | 1302 | G | A | 38.46% | 15 | 0 | - | 0 |
| **1350** | 1348 | A | G | 23.07% | - | 0 | 9 | 0 |
| **1362** | 1360 | G | A | 84.61% | 33 | 0 | - | 0 |
| **1370** | 1368 | G | T | 23.07% | 0 | 0 | - | 9 |
| **1385** | 1383 | T | C | 10.25% | 0 | 4 | 0 | - |
| **1417** | 1415 | C | T | 17.94% | 1 | - | 0 | 6 |

**Table S4.** Distance scores calculated using 40 Asia isolates by SeqMan Pro™ software

| | 1 | 2 | 3 | 4 | 5 | 6 | 7 | 8 | 9 | 10 | 11 | 12 | 13 | 14 | 15 | 16 | 17 | 18 | 19 | 20 | 21 | 22 | 23 | 24 | 25 | 26 | 27 | 28 | 29 | 30 | 31 | 32 | 33 | 34 | 35 | 36 | 37 | 38 | 39 | 40 |
|---|---|---|---|---|---|---|---|---|---|---|---|---|---|---|---|---|---|---|---|---|---|---|---|---|---|---|---|---|---|---|---|---|---|---|---|---|---|---|---|---|
| **XJ-13:China:2013** | 0.00 | 0.00 | 0.00 | 0.00 | 0.00 | 0.01 | 0.01 | 0.01 | 0.05 | 0.05 | 0.05 | 0.05 | 0.05 | 0.05 | 0.06 | 0.06 | 0.07 | 0.05 | 0.04 | 0.04 | 0.04 | 0.05 | 0.06 | 0.03 | 0.03 | 0.03 | 0.03 | 0.05 | 0.04 | 0.03 | 0.03 | 0.03 | 0.03 | 0.04 | 0.04 | 0.01 | 0.01 | 0.01 | 0.01 | 0.00 |
| **SD-12:China:2012** | 0.00 | 0.00 | 0.00 | 0.00 | 0.00 | 0.01 | 0.01 | 0.01 | 0.05 | 0.05 | 0.05 | 0.05 | 0.05 | 0.05 | 0.06 | 0.06 | 0.07 | 0.05 | 0.04 | 0.04 | 0.04 | 0.05 | 0.06 | 0.03 | 0.03 | 0.03 | 0.03 | 0.05 | 0.04 | 0.04 | 0.03 | 0.03 | 0.04 | 0.04 | 0.04 | 0.01 | 0.01 | 0.01 | 0.01 | 0.00 |
| **GS-12:China:2012** | 0.00 | 0.00 | 0.00 | 0.00 | 0.00 | 0.01 | 0.01 | 0.01 | 0.05 | 0.05 | 0.05 | 0.05 | 0.05 | 0.05 | 0.06 | 0.06 | 0.07 | 0.05 | 0.04 | 0.04 | 0.04 | 0.05 | 0.06 | 0.03 | 0.03 | 0.03 | 0.03 | 0.05 | 0.04 | 0.03 | 0.03 | 0.03 | 0.03 | 0.04 | 0.04 | 0.01 | 0.01 | 0.01 | 0.01 | 0.00 |
| **YN14:China:2014** | 0.00 | 0.00 | 0.00 | 0.00 | 0.00 | 0.01 | 0.02 | 0.02 | 0.05 | 0.05 | 0.05 | 0.05 | 0.05 | 0.05 | 0.06 | 0.07 | 0.07 | 0.05 | 0.04 | 0.05 | 0.05 | 0.06 | 0.06 | 0.03 | 0.04 | 0.04 | 0.03 | 0.05 | 0.04 | 0.04 | 0.03 | 0.03 | 0.04 | 0.04 | 0.04 | 0.01 | 0.02 | 0.01 | 0.01 | 0.00 |
| **YN-13:China:2013** | 0.00 | 0.00 | 0.00 | 0.00 | 0.00 | 0.01 | 0.01 | 0.01 | 0.05 | 0.05 | 0.05 | 0.05 | 0.05 | 0.05 | 0.06 | 0.06 | 0.07 | 0.05 | 0.04 | 0.04 | 0.04 | 0.05 | 0.06 | 0.03 | 0.03 | 0.03 | 0.03 | 0.05 | 0.04 | 0.04 | 0.03 | 0.03 | 0.04 | 0.04 | 0.04 | 0.01 | 0.01 | 0.01 | 0.01 | 0.00 |
| **HLJ12:China:2012** | 0.01 | 0.01 | 0.01 | 0.01 | 0.01 | 0.00 | 0.01 | 0.01 | 0.05 | 0.05 | 0.05 | 0.05 | 0.05 | 0.05 | 0.05 | 0.06 | 0.07 | 0.04 | 0.04 | 0.04 | 0.04 | 0.05 | 0.05 | 0.03 | 0.03 | 0.03 | 0.03 | 0.05 | 0.04 | 0.03 | 0.03 | 0.02 | 0.03 | 0.04 | 0.04 | 0.01 | 0.01 | 0.01 | 0.01 | 0.01 |
| **XJ14a:China:2014** | 0.01 | 0.01 | 0.01 | 0.02 | 0.01 | 0.01 | 0.00 | 0.01 | 0.05 | 0.05 | 0.05 | 0.05 | 0.05 | 0.05 | 0.06 | 0.07 | 0.07 | 0.05 | 0.04 | 0.04 | 0.04 | 0.06 | 0.06 | 0.03 | 0.03 | 0.03 | 0.03 | 0.05 | 0.04 | 0.03 | 0.03 | 0.03 | 0.03 | 0.04 | 0.04 | 0.01 | 0.01 | 0.01 | 0.01 | 0.01 |
| **XJ16a:China:2016** | 0.01 | 0.01 | 0.01 | 0.02 | 0.01 | 0.01 | 0.01 | 0.00 | 0.05 | 0.05 | 0.05 | 0.05 | 0.05 | 0.05 | 0.06 | 0.07 | 0.07 | 0.05 | 0.04 | 0.04 | 0.04 | 0.06 | 0.06 | 0.03 | 0.03 | 0.03 | 0.03 | 0.05 | 0.04 | 0.04 | 0.03 | 0.03 | 0.03 | 0.04 | 0.04 | 0.01 | 0.02 | 0.01 | 0.01 | 0.01 |
| **HV7601:Japan:2005** | 0.05 | 0.05 | 0.05 | 0.05 | 0.05 | 0.05 | 0.05 | 0.05 | 0.00 | 0.01 | 0.01 | 0.01 | 0.00 | 0.01 | 0.02 | 0.03 | 0.04 | 0.02 | 0.02 | 0.02 | 0.03 | 0.04 | 0.04 | 0.04 | 0.04 | 0.04 | 0.04 | 0.05 | 0.05 | 0.05 | 0.04 | 0.04 | 0.04 | 0.05 | 0.05 | 0.05 | 0.05 | 0.04 | 0.05 | 0.05 |
| **KoMo71:Japan:1971** | 0.05 | 0.05 | 0.05 | 0.05 | 0.05 | 0.05 | 0.05 | 0.05 | 0.01 | 0.00 | 0.01 | 0.01 | 0.01 | 0.02 | 0.02 | 0.04 | 0.04 | 0.03 | 0.02 | 0.03 | 0.03 | 0.04 | 0.05 | 0.05 | 0.05 | 0.05 | 0.04 | 0.06 | 0.06 | 0.05 | 0.05 | 0.04 | 0.05 | 0.06 | 0.06 | 0.05 | 0.05 | 0.05 | 0.05 | 0.05 |
| **RtNag82:Japan:1982** | 0.05 | 0.05 | 0.05 | 0.05 | 0.05 | 0.05 | 0.05 | 0.05 | 0.01 | 0.01 | 0.00 | 0.01 | 0.01 | 0.01 | 0.02 | 0.04 | 0.04 | 0.02 | 0.02 | 0.02 | 0.03 | 0.04 | 0.04 | 0.04 | 0.05 | 0.05 | 0.04 | 0.06 | 0.05 | 0.05 | 0.04 | 0.04 | 0.04 | 0.05 | 0.05 | 0.05 | 0.05 | 0.05 | 0.05 | 0.05 |
| **ChAb76:Japan:1976** | 0.05 | 0.05 | 0.05 | 0.05 | 0.05 | 0.05 | 0.05 | 0.05 | 0.01 | 0.01 | 0.01 | 0.00 | 0.00 | 0.01 | 0.02 | 0.04 | 0.04 | 0.02 | 0.02 | 0.02 | 0.03 | 0.04 | 0.04 | 0.04 | 0.05 | 0.05 | 0.04 | 0.06 | 0.05 | 0.05 | 0.04 | 0.04 | 0.04 | 0.05 | 0.05 | 0.05 | 0.05 | 0.04 | 0.05 | 0.05 |
| **ChYu78:Japan:1978** | 0.05 | 0.05 | 0.05 | 0.05 | 0.05 | 0.05 | 0.05 | 0.05 | 0.00 | 0.01 | 0.01 | 0.00 | 0.00 | 0.01 | 0.02 | 0.03 | 0.04 | 0.02 | 0.02 | 0.02 | 0.03 | 0.04 | 0.04 | 0.04 | 0.04 | 0.04 | 0.04 | 0.06 | 0.05 | 0.05 | 0.04 | 0.04 | 0.04 | 0.05 | 0.05 | 0.05 | 0.05 | 0.04 | 0.05 | 0.05 |
| **RtNag76:Japan:1976** | 0.05 | 0.05 | 0.05 | 0.05 | 0.05 | 0.05 | 0.05 | 0.05 | 0.01 | 0.02 | 0.01 | 0.01 | 0.01 | 0.00 | 0.02 | 0.04 | 0.05 | 0.03 | 0.02 | 0.03 | 0.03 | 0.04 | 0.04 | 0.04 | 0.04 | 0.04 | 0.04 | 0.06 | 0.06 | 0.05 | 0.05 | 0.04 | 0.04 | 0.05 | 0.05 | 0.05 | 0.05 | 0.05 | 0.05 | 0.05 |
| **TV8725:Japan:1988** | 0.06 | 0.06 | 0.06 | 0.06 | 0.06 | 0.05 | 0.06 | 0.06 | 0.02 | 0.02 | 0.02 | 0.02 | 0.02 | 0.02 | 0.00 | 0.04 | 0.05 | 0.03 | 0.03 | 0.03 | 0.04 | 0.05 | 0.05 | 0.05 | 0.05 | 0.05 | 0.05 | 0.06 | 0.06 | 0.05 | 0.05 | 0.05 | 0.05 | 0.06 | 0.06 | 0.06 | 0.06 | 0.05 | 0.06 | 0.06 |
| **RtShiz06s:Japan:2006** | 0.06 | 0.06 | 0.06 | 0.07 | 0.06 | 0.06 | 0.07 | 0.07 | 0.03 | 0.04 | 0.04 | 0.04 | 0.03 | 0.04 | 0.04 | 0.00 | 0.03 | 0.04 | 0.04 | 0.05 | 0.05 | 0.06 | 0.06 | 0.06 | 0.06 | 0.06 | 0.06 | 0.07 | 0.07 | 0.06 | 0.06 | 0.05 | 0.06 | 0.07 | 0.07 | 0.06 | 0.06 | 0.06 | 0.06 | 0.06 |
| **NT1203:Japan:2012** | 0.07 | 0.07 | 0.07 | 0.07 | 0.07 | 0.07 | 0.07 | 0.07 | 0.04 | 0.04 | 0.04 | 0.04 | 0.04 | 0.05 | 0.05 | 0.03 | 0.00 | 0.05 | 0.05 | 0.05 | 0.06 | 0.06 | 0.07 | 0.07 | 0.07 | 0.07 | 0.07 | 0.08 | 0.08 | 0.07 | 0.07 | 0.06 | 0.07 | 0.08 | 0.08 | 0.07 | 0.08 | 0.07 | 0.07 | 0.07 |
| **AyTochi86:Japan:1986** | 0.05 | 0.05 | 0.05 | 0.05 | 0.05 | 0.04 | 0.05 | 0.05 | 0.02 | 0.03 | 0.02 | 0.02 | 0.02 | 0.03 | 0.03 | 0.04 | 0.05 | 0.00 | 0.01 | 0.02 | 0.02 | 0.04 | 0.04 | 0.04 | 0.04 | 0.04 | 0.04 | 0.06 | 0.05 | 0.04 | 0.04 | 0.03 | 0.04 | 0.05 | 0.05 | 0.05 | 0.05 | 0.05 | 0.05 | 0.05 |
| **RtToya80:Japan:1980** | 0.04 | 0.04 | 0.04 | 0.04 | 0.04 | 0.04 | 0.04 | 0.04 | 0.02 | 0.02 | 0.02 | 0.02 | 0.02 | 0.02 | 0.03 | 0.04 | 0.05 | 0.01 | 0.00 | 0.01 | 0.02 | 0.03 | 0.03 | 0.03 | 0.03 | 0.03 | 0.03 | 0.05 | 0.04 | 0.04 | 0.03 | 0.03 | 0.03 | 0.04 | 0.04 | 0.04 | 0.04 | 0.04 | 0.04 | 0.04 |
| **RtTochi86:Japan:1986** | 0.04 | 0.04 | 0.04 | 0.05 | 0.04 | 0.04 | 0.04 | 0.04 | 0.02 | 0.03 | 0.02 | 0.02 | 0.02 | 0.03 | 0.03 | 0.05 | 0.05 | 0.02 | 0.01 | 0.00 | 0.01 | 0.03 | 0.03 | 0.03 | 0.04 | 0.04 | 0.03 | 0.05 | 0.04 | 0.04 | 0.03 | 0.03 | 0.04 | 0.04 | 0.04 | 0.04 | 0.05 | 0.04 | 0.04 | 0.04 |
| **RtPy91:South Korea** | 0.04 | 0.04 | 0.04 | 0.05 | 0.04 | 0.04 | 0.04 | 0.04 | 0.03 | 0.03 | 0.03 | 0.03 | 0.03 | 0.03 | 0.04 | 0.05 | 0.06 | 0.02 | 0.02 | 0.01 | 0.00 | 0.02 | 0.02 | 0.03 | 0.04 | 0.04 | 0.03 | 0.05 | 0.05 | 0.04 | 0.04 | 0.03 | 0.04 | 0.04 | 0.04 | 0.04 | 0.04 | 0.04 | 0.04 | 0.04 |
| **RtJe00:South Korea** | 0.05 | 0.05 | 0.05 | 0.06 | 0.05 | 0.05 | 0.06 | 0.06 | 0.04 | 0.04 | 0.04 | 0.04 | 0.04 | 0.04 | 0.05 | 0.06 | 0.06 | 0.04 | 0.03 | 0.03 | 0.02 | 0.00 | 0.04 | 0.05 | 0.05 | 0.05 | 0.05 | 0.06 | 0.06 | 0.05 | 0.05 | 0.05 | 0.05 | 0.06 | 0.06 | 0.06 | 0.06 | 0.05 | 0.06 | 0.05 |

| | | | | | | | | | | | | | | | | | | | | | | | | | | | | | | | | | | | | | | | | |
|---|---|---|---|---|---|---|---|---|---|---|---|---|---|---|---|---|---|---|---|---|---|---|---|---|---|---|---|---|---|---|---|---|---|---|---|---|---|---|---|---|
| **PcOj11:South Korea** | 0.06 | 0.06 | 0.06 | 0.06 | 0.06 | 0.05 | 0.06 | 0.06 | 0.04 | 0.05 | 0.04 | 0.04 | 0.04 | 0.04 | 0.05 | 0.06 | 0.07 | 0.04 | 0.03 | 0.03 | 0.02 | 0.04 | 0.00 | 0.05 | 0.05 | 0.05 | 0.05 | 0.07 | 0.06 | 0.05 | 0.05 | 0.05 | 0.05 | 0.06 | 0.06 | 0.05 | 0.06 | 0.05 | 0.06 | 0.05 |
| **RtAichi06a:Japan:2006** | 0.03 | 0.03 | 0.03 | 0.03 | 0.03 | 0.03 | 0.03 | 0.03 | 0.04 | 0.05 | 0.04 | 0.04 | 0.04 | 0.04 | 0.05 | 0.06 | 0.07 | 0.04 | 0.03 | 0.03 | 0.03 | 0.05 | 0.05 | 0.00 | 0.02 | 0.02 | 0.02 | 0.04 | 0.03 | 0.03 | 0.02 | 0.02 | 0.02 | 0.03 | 0.03 | 0.03 | 0.03 | 0.03 | 0.03 | 0.03 |
| **TV0004:Japan:2000** | 0.03 | 0.03 | 0.03 | 0.04 | 0.03 | 0.03 | 0.03 | 0.03 | 0.04 | 0.05 | 0.05 | 0.05 | 0.04 | 0.04 | 0.05 | 0.06 | 0.07 | 0.04 | 0.03 | 0.04 | 0.04 | 0.05 | 0.05 | 0.02 | 0.00 | 0.00 | 0.02 | 0.04 | 0.03 | 0.03 | 0.02 | 0.02 | 0.02 | 0.03 | 0.03 | 0.03 | 0.03 | 0.03 | 0.03 | 0.03 |
| **TV9923:Japan:2000** | 0.03 | 0.03 | 0.03 | 0.04 | 0.03 | 0.03 | 0.03 | 0.03 | 0.04 | 0.05 | 0.05 | 0.05 | 0.04 | 0.04 | 0.05 | 0.06 | 0.07 | 0.04 | 0.03 | 0.04 | 0.04 | 0.05 | 0.05 | 0.02 | 0.00 | 0.00 | 0.02 | 0.04 | 0.03 | 0.03 | 0.02 | 0.02 | 0.02 | 0.03 | 0.03 | 0.03 | 0.03 | 0.03 | 0.03 | 0.03 |
| **RtNag96:Japan:1996** | 0.03 | 0.03 | 0.03 | 0.03 | 0.03 | 0.03 | 0.03 | 0.03 | 0.04 | 0.04 | 0.04 | 0.04 | 0.04 | 0.04 | 0.05 | 0.06 | 0.07 | 0.04 | 0.03 | 0.03 | 0.03 | 0.05 | 0.05 | 0.02 | 0.02 | 0.02 | 0.00 | 0.03 | 0.02 | 0.01 | 0.01 | 0.01 | 0.01 | 0.03 | 0.03 | 0.03 | 0.03 | 0.03 | 0.03 | 0.03 |
| **SNU1:South Korea** | 0.05 | 0.05 | 0.05 | 0.05 | 0.05 | 0.05 | 0.05 | 0.05 | 0.05 | 0.06 | 0.06 | 0.06 | 0.06 | 0.06 | 0.06 | 0.07 | 0.08 | 0.06 | 0.05 | 0.05 | 0.05 | 0.06 | 0.07 | 0.04 | 0.04 | 0.04 | 0.03 | 0.00 | 0.02 | 0.02 | 0.02 | 0.03 | 0.03 | 0.05 | 0.05 | 0.05 | 0.05 | 0.05 | 0.05 | 0.05 |
| **RtGoH14:South Korea** | 0.04 | 0.04 | 0.04 | 0.04 | 0.04 | 0.04 | 0.04 | 0.04 | 0.05 | 0.06 | 0.05 | 0.05 | 0.05 | 0.06 | 0.06 | 0.07 | 0.08 | 0.05 | 0.04 | 0.04 | 0.05 | 0.06 | 0.06 | 0.03 | 0.03 | 0.03 | 0.02 | 0.02 | 0.00 | 0.01 | 0.02 | 0.02 | 0.03 | 0.04 | 0.04 | 0.04 | 0.04 | 0.04 | 0.04 | 0.04 |
| **PcKw11:South Korea** | 0.03 | 0.04 | 0.03 | 0.04 | 0.04 | 0.03 | 0.03 | 0.04 | 0.05 | 0.05 | 0.05 | 0.05 | 0.05 | 0.05 | 0.05 | 0.06 | 0.07 | 0.04 | 0.04 | 0.04 | 0.04 | 0.05 | 0.05 | 0.03 | 0.03 | 0.03 | 0.01 | 0.02 | 0.01 | 0.00 | 0.01 | 0.01 | 0.02 | 0.04 | 0.04 | 0.03 | 0.03 | 0.04 | 0.03 | 0.03 |
| **RtUi02:South Korea** | 0.03 | 0.03 | 0.03 | 0.03 | 0.03 | 0.03 | 0.03 | 0.03 | 0.04 | 0.05 | 0.04 | 0.04 | 0.04 | 0.05 | 0.05 | 0.06 | 0.07 | 0.04 | 0.03 | 0.03 | 0.04 | 0.05 | 0.05 | 0.02 | 0.02 | 0.02 | 0.01 | 0.02 | 0.02 | 0.01 | 0.00 | 0.01 | 0.01 | 0.03 | 0.03 | 0.03 | 0.03 | 0.03 | 0.03 | 0.03 |
| **RtGu01:South Korea** | 0.03 | 0.03 | 0.03 | 0.03 | 0.03 | 0.02 | 0.03 | 0.03 | 0.04 | 0.04 | 0.04 | 0.04 | 0.04 | 0.04 | 0.05 | 0.05 | 0.06 | 0.03 | 0.03 | 0.03 | 0.03 | 0.05 | 0.05 | 0.02 | 0.02 | 0.02 | 0.01 | 0.03 | 0.02 | 0.01 | 0.01 | 0.00 | 0.01 | 0.03 | 0.03 | 0.02 | 0.03 | 0.03 | 0.03 | 0.03 |
| **ChYa07:South Korea** | 0.03 | 0.04 | 0.03 | 0.04 | 0.04 | 0.03 | 0.03 | 0.03 | 0.04 | 0.05 | 0.04 | 0.04 | 0.04 | 0.04 | 0.05 | 0.06 | 0.07 | 0.04 | 0.03 | 0.04 | 0.04 | 0.05 | 0.05 | 0.02 | 0.02 | 0.02 | 0.01 | 0.03 | 0.03 | 0.02 | 0.01 | 0.01 | 0.00 | 0.04 | 0.04 | 0.03 | 0.03 | 0.03 | 0.03 | 0.03 |
| **GM1404:Japan:2014** | 0.04 | 0.04 | 0.04 | 0.04 | 0.04 | 0.04 | 0.04 | 0.04 | 0.05 | 0.06 | 0.05 | 0.05 | 0.05 | 0.05 | 0.06 | 0.07 | 0.08 | 0.05 | 0.04 | 0.04 | 0.04 | 0.06 | 0.06 | 0.03 | 0.03 | 0.03 | 0.03 | 0.05 | 0.04 | 0.04 | 0.03 | 0.03 | 0.04 | 0.00 | 0.01 | 0.04 | 0.04 | 0.04 | 0.04 | 0.04 |
| **NT1101:Japan:2011** | 0.04 | 0.04 | 0.04 | 0.04 | 0.04 | 0.04 | 0.04 | 0.04 | 0.05 | 0.06 | 0.05 | 0.05 | 0.05 | 0.05 | 0.06 | 0.07 | 0.08 | 0.05 | 0.04 | 0.04 | 0.04 | 0.06 | 0.06 | 0.03 | 0.03 | 0.03 | 0.03 | 0.05 | 0.04 | 0.04 | 0.03 | 0.03 | 0.04 | 0.01 | 0.00 | 0.04 | 0.04 | 0.04 | 0.04 | 0.04 |
| **BJ1401:China:2014** | 0.01 | 0.01 | 0.01 | 0.01 | 0.01 | 0.01 | 0.01 | 0.01 | 0.05 | 0.05 | 0.05 | 0.05 | 0.05 | 0.05 | 0.06 | 0.06 | 0.07 | 0.05 | 0.04 | 0.04 | 0.04 | 0.06 | 0.05 | 0.03 | 0.03 | 0.03 | 0.03 | 0.05 | 0.04 | 0.03 | 0.03 | 0.02 | 0.03 | 0.04 | 0.04 | 0.00 | 0.00 | 0.01 | 0.01 | 0.01 |
| **SC15:China:2015** | 0.01 | 0.01 | 0.01 | 0.02 | 0.01 | 0.01 | 0.01 | 0.02 | 0.05 | 0.05 | 0.05 | 0.05 | 0.05 | 0.05 | 0.06 | 0.06 | 0.08 | 0.05 | 0.04 | 0.05 | 0.04 | 0.06 | 0.06 | 0.03 | 0.03 | 0.03 | 0.03 | 0.05 | 0.04 | 0.03 | 0.03 | 0.03 | 0.03 | 0.04 | 0.04 | 0.00 | 0.00 | 0.01 | 0.01 | 0.01 |
| **Sn1203:China:2012** | 0.01 | 0.01 | 0.01 | 0.01 | 0.01 | 0.01 | 0.01 | 0.01 | 0.04 | 0.05 | 0.05 | 0.04 | 0.04 | 0.05 | 0.05 | 0.06 | 0.07 | 0.05 | 0.04 | 0.04 | 0.04 | 0.05 | 0.05 | 0.03 | 0.03 | 0.03 | 0.03 | 0.05 | 0.04 | 0.04 | 0.03 | 0.03 | 0.03 | 0.04 | 0.04 | 0.01 | 0.01 | 0.00 | 0.01 | 0.01 |
| **LN14dd:China:2014** | 0.01 | 0.01 | 0.01 | 0.01 | 0.01 | 0.01 | 0.01 | 0.01 | 0.05 | 0.05 | 0.05 | 0.05 | 0.05 | 0.05 | 0.06 | 0.06 | 0.07 | 0.05 | 0.04 | 0.04 | 0.04 | 0.06 | 0.06 | 0.03 | 0.03 | 0.03 | 0.03 | 0.05 | 0.04 | 0.03 | 0.03 | 0.03 | 0.03 | 0.04 | 0.04 | 0.01 | 0.01 | 0.01 | 0.00 | 0.01 |
| **LN-12:China:2012** | 0.00 | 0.00 | 0.00 | 0.00 | 0.00 | 0.01 | 0.01 | 0.01 | 0.05 | 0.05 | 0.05 | 0.05 | 0.05 | 0.05 | 0.06 | 0.06 | 0.07 | 0.05 | 0.04 | 0.04 | 0.04 | 0.05 | 0.05 | 0.03 | 0.03 | 0.03 | 0.03 | 0.05 | 0.04 | 0.03 | 0.03 | 0.03 | 0.03 | 0.04 | 0.04 | 0.01 | 0.01 | 0.01 | 0.01 | 0.00 |

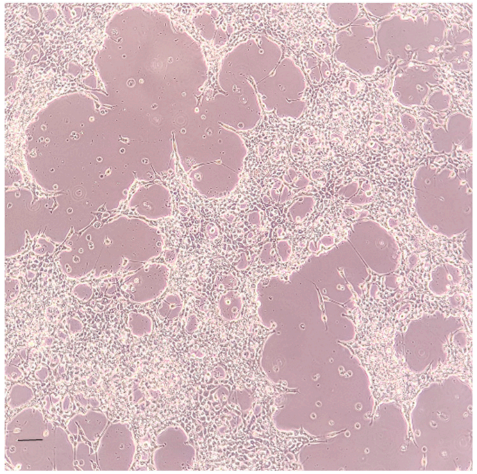

**Figure S1.** CPE result of EPC cell line after 48h infection with IHN virus strain SNU1. (Bar indicating 50 μm).
